# Enhanced OH^−^ Transport Properties of Bio-Based Anion-Exchange Membranes for Different Applications

**DOI:** 10.3390/membranes15080229

**Published:** 2025-07-31

**Authors:** Suer Kurklu-Kocaoglu, Daniela Ramírez-Espinosa, Clara Casado-Coterillo

**Affiliations:** 1 Department of Chemical and Biomolecular Engineering, Universidad de Cantabria, A. Los Castros s/n, 39005 Santander, Spain; casadoc@unican.es; 2 Department of Chemical Engineering, Pamukkale University, Pamukkale, 20160 Denizli, Turkey; 3 McKetta Department of Chemical Engineering, The University of Texas at Austin, Austin, TX 78712, USA; 4 Decarbonization Group, Instituto Tecnológico del Plástico (AIMPLAS), C/Gustave Eiffel 4, 46980 Paterna, Valencia, Spain; daramirez@aimplas.es

**Keywords:** a anion exchange membranes, chitosan, polyvinyl alcohol, mixedmatrix membranes, ZnO, POP

## Abstract

The demand for anion exchange membranes (AEMs) is growing due to their applications in water electrolysis, CO_2_ reduction conversion and fuel cells, as well as water treatment, driven by the increasing energy demand and the need for a sustainable future. However, current AEMs still face challenges, such as insufficient permeability and stability in strongly acidic or alkaline media, which limit their durability and the sustainability of membrane fabrication. In this study, polyvinyl alcohol (PVA) and chitosan (CS) biopolymers are selected for membrane preparation. Zinc oxide (ZnO) and porous organic polymer (POP) nanoparticles are also introduced within the PVA-CS polymer blends to make mixed-matrix membranes (MMMs) with increased OH^−^ transport sites. The membranes are characterized based on typical properties for AEM applications, such as thickness, water uptake, KOH uptake, Cl^−^ and OH^−^ permeability and ion exchange capacity (IEC). The OH^−^ transport of the PVA-CS blend is increased by at least 94.2% compared with commercial membranes. The incorporation of non-porous ZnO and porous POP nanoparticles into the polymer blend does not compromise the OH^−^ transport properties. On the contrary, ZnO nanoparticles enhance the membrane’s water retention capacity, provide basic surface sites that facilitate hydroxide ion conduction and reinforce the mechanical and thermal stability. In parallel, POPs introduce a highly porous architecture that increases the internal surface area and promotes the formation of continuous hydrated pathways, essential to efficient OH^−^ mobility. Furthermore, the presence of POPs also contributes to reinforcing the mechanical integrity of the membrane. Thus, PVA-CS bio-based membranes are a promising alternative to conventional ion exchange membranes for various applications.

## 1. Introduction

Fossil fuels have long been the primary source of energy, but the ongoing transition to cleaner sources to alleviate the stress upon climate and the environment has turned the attention of researchers to other energy sources [1]. Green hydrogen production is significant for this energy transition, supporting energy storage and the development of sustainable fuels. Alkaline water electrolysis, a technology that was developed more than a century ago, could be adapted with new materials to enable hydrogen production [2]. Although membranes for alkaline electrochemical devices are often taken for granted in the development of CO_2_ electrolyzers and energy storage applications [1,3,4], natural polymers from plant or marine resources have attracted much attention in the last decade [5,6,7]. Gel polymer electrolytes have been analyzed as sustainable ion exchange separators improving the OH^−^ transport of the whole device [8,9]. Polyvinyl alcohol (PVA) is a low-cost, synthetic, biodegradable polymer used largely in electrochemical applications, providing hydrophilicity and mechanical properties to other materials [10]. However, its anion conductivity is low, so blends with ion-exchangeable polymers and other materials are desirable [11]. Chitosan (CS) is a polysaccharide obtained from the deacetylation of chitin, the second most abundantly encountered natural polymer, in alkaline solutions. CS is widely studied as an alternative material for diverse membrane applications due to low toxicity, acceptable biodegradability and biocompatibility, film-forming properties, hydrophilicity, chemical resistance in alkaline medium and easy functionalization [12,13,14,15,16]. The hydrophilicity may lower the mechanical strength, but the presence of OH and NH_2_ functional groups in the polymer backbone allows for the blending with other polymers like PVA or other materials such as organic liquids, e.g., poly(ethylene glycol) (PEG) or ionic liquids (ILs), or inorganic porous particles (zeolites, silica, carbon based, etc.) to improve performance in various applications, such as batteries, electrochemical CO_2_ reduction (CO_2_RR) and water treatment [17,18,19,20,21,22].

Carboxymethyl cellulose (CMC) is an additive used as a potential green polymer electrolyte in battery applications, alongside polyethylene oxide (PEO) and CS [13,23]. Even though the main goal is to develop sustainable batteries, studies have shown that the addition of Na-CMC improves mechanical properties, while the chemical modification of CS with CMC results in higher ionic conductivity. Both improvements in mechanical properties and ionic conductivity are critical to battery performance [3].

Other studies use chitosan and polyvinyl alcohol to enhance the mechanical properties and ionic conductivity of the separator and enhance battery performance, inspired by other applications of PVA and CS in water treatment and electrolyzers. The preparation method, characterization and applications are summarized in Table 1. Overall, these studies show improved mechanical properties and ionic conductivity of AEMs.

Both Qiao et al. and Santos et al. studied PVA solid polymer electrolyte performance by doping KOH in the polymer electrolyte. Polyvinylpyrrolidone (PVP) is used to enhance the oxidative stability and chemical stability of PVA under alkaline conditions. Doping PVA or the PVA/PVP blend resulted in increased ionic conductivity, and the addition of PVP led to high alkaline stability [9,24]. Gopi et al. systematically studied PVA-CS membranes in different amounts for fuel cell application. They further crosslinked PVA with quaternization agents (QPVA), and the QPVA-CS membranes demonstrated enhanced alkaline stability, lasting up to 300 h in 4 M KOH solution at 80 °C [14].

To further improve solid polymer electrolytes, PVA and CS are further hybridized by the addition of various fillers to create a mixed-matrix membrane (MMM). Permana et al. investigated PVA-CS blends as a promising solution to enhance the conductivity and mechanical properties of CS ion exchange membranes, owing to the strong hydrogen bonding between the OH groups of PVA and the OH and NH_2_ groups of CS [25]. Blending CS with PVA results in increased water uptake, particularly as the PVA content rises due to the hydroxyl groups in PVA. High water uptake could lead to higher permeability. However, CS-PVA membranes with hematite did not show a clear trend with PVA content. They addressed the low conductivity of CS membranes and increased the conductivity by in situ formation of hematite (Fe_2_O_3_). CS-PVA-H3070 membranes showed the highest ion exchange capacity (IEC) and proton conductivity of 4300 meq/g and 6.71 × 10^−2^ S·cm^−1^, respectively.

Garcia-Cruz et al. studied CS-based MMMs to study the feasibility of new materials and investigate the performance of membranes in alkaline electrosynthesis. They used various fillers, such as IL [emim][OAc], metallic Sn powder, layered titanosilicate AM-4 and layered stannosilicate UZAR-S3, to enhance the ionic conductivity and stability of the membranes. Among these fillers, the addition of tin to CS resulted in higher conductivity than that of the bare CS membrane [26]. Overall, PVA and CS membranes exhibit properties regarding ionic conductivity, ion exchange capacity (IEC), mechanical strength, and thermal and alkaline stability relevant to alkaline and filtration applications, with values of an order of magnitude approaching conventional ion exchange membrane materials. All these are summarized in Table 1. It is noteworthy that the authors usually conclude that the most relevant properties are a combination of several others, often leading to structure–property correlations or trade-offs to be taken into account to predict membrane performance. This correlation can also be of use for the characterization of new membrane materials at laboratory scale.

**Table 1:** Biopolymer-based anion exchange membranes, properties and applications reported in the literature.

Membrane	Preparation Method	Characterization	Application	Relevant Properties	Ref.
Na-CMC/PEO	Dip coating on PEO supports	Thermal stability (N_2_), FESEM, EIS, Na transference number, interfacial properties, LSV and galvanostatic charge (Na^+^ deinsertion)/discharge (Na^+^ insertion)	Na-ion batteries	Mechanical resistance as dimensional stability	[23]
CMC chitosan membrane	Solution casting	ATR-FTIR, ^1^H NMR, EA, XRD, EIS and SEM	Development of alternative membrane materials not petroleum-related	Ionic conductivity (0.37 mS at 60 °C) Interaction polymer solvent (acetic acid) by ATR-FTIR	[13]
PVA-CS membrane	Blend and solution casting	EIS, LSV and TNM	Li-ion batteries	Ionic conductivity (0.84 mS/cm) vs. ion transport	[19]
PVA-KOH hydrogels	Solution casting	OCV (using 6 M KOH), WU, XRD, TGA, XPS, ATR-FTIR,	Zn–air batteries	Functionalization to avoid ZnO precipitation—stability	[27]
CS-PVA-Fe_2_O_3_	Solution casting and soaked in iron sand leachate	XRD, FTIR, SEM WU, tensile testing, IEC, methanol permeability and proton conductivity	DMFC	relationship among IEC, ionic conductivity and alcohol crossover	[25]
CS-PVA-based mixed-matrix membranes	Blending	SEM, XPS, XRD, TGA, WU, IEC, specific conductivity, alcohol permeability and PEM reactor performance	Organic electrosynthesis in alkaline media	Correlation among IEC, anion conductivity and crossover	[11]
MA-chitosan membrane	Blending	TGA, WU, SD, IEC and EIS (H^+^)	PEMFC	IEC and proton conductivity correlation	[15]
PVA-CS AEM	Solution casting, quaternized or not	TGA, FTIR, EIS and alkaline stability	AEMFC	Alkaline stability reduced ionic conductivity loss with time	[14]
PVA/PVP AEM	Solution casting	FTIR, TGA, SEM, EIS, KOH uptake, SEM	AEMFC	KOH uptake increased membrane stability	[24]

CMC: carboxymethyl cellulose; EA: elemental analysis; LSV: Linear Sweep Voltammetry; MA: Maleic Anhydride; OCV: Open Circuit Voltage; PEO: Polyethylene oxide; PVA: polyvinyl alcohol; WU: water uptake; TNM: transference number analysis.

In the present study, a simple way of producing ion echange membranes based on PVA and CS biopolymer blends under ambient conditions is presented. Both biopolymers are dissolved individually and blended in different ratios. The optimal blend is further hybridized by non-porous and porous fillers to observe the effect on the structural, chemical and thermal stability of the membranes, investigated by monitoring different properties. Water and ion uptake and transport are characterized by water uptake, ion exchange capacity and permeability.

## 2. Materials and Methods

Acetic acid glacial (Reag. Ph. Eur.), sodium chloride for analysis (NaCl, 99.5%, PA-ACS-ISO) and phenolphthalein solution (1%) were supplied by Panreac Quimica (Barcelona, Spain). Chitosan (CS; coarse ground powder and flakes, MW = 310,000–375,000 and deacetylation degree 75%, based on the viscosity range 800–2000 mPa s) and poly (vinyl alcohol) (PVA; MW 85,000–124,000, 99+% hydrolyzed) were purchased from Aldrich Chemistry (Madrid, Spain). Potassium hydroxide (KOH; analytical reagent grade) and hydrochloride acid (HCl; 1 M stock solution) were purchased from Fisher Scientific (Loughborough, UK). Silver nitrate (AgNO_3_; ACS reagent, ≥99%), potassium hydrogen phthalate (KHP; BioXtra, ≥99.5%), zinc oxide (<100 nm particle size) and S.L. NaOH (pellets) were supplied by Sigma Aldrich (St. Louis, MI, USA). K_2_CrO_4_ (5% *w*/*v*, EssentQ) was purchased from Scharlab (Barcelona, Spain). All chemicals were used as received without further purification. Porous organic polymer (POP-1), used as a highly porous nanofiller, was provided by the Institute of Polymer Science and Technology (CSIC, Madrid, Spain), characterized in a prior work [28]. Celgard 3401 was kindly supplied by Celgard (AsahiKasei Group, Cary, NC, USA), and Sustainion membranes were purchased from Dioxide Materials (Boca Raton, FL, USA).

### 2.1. Membrane Preparation

A polyvinyl alcohol solution (PVA) (4 wt.%) was dissolved in ultrapure water and stirred at 80 °C under reflux for 1 day until a clear solution was obtained. CS solutions of 2 wt.% and 4 wt.% were prepared in 2 wt.% acetic acid aqueous solution. Both CS solutions were stirred for 24 h. The 4 wt.% CS solution was used for CS membranes, while CS-PVA blend membranes were prepared by using 2 wt.% CS solutions. The viscosity values of the 2% and 4% CS solutions were 3419 and 7742 cP, respectively [29]. Different ratios of PVA and CS solutions were mixed and stirred for one day to obtain a homogeneous solution. The predetermined amount of CS or PVA-CS solution was cast on a Petri dish having a diameter of around 7 cm. After casting, all membranes were dried in the fume hood under ambient conditions until they could be peeled off from the Petri dish. It took 4–5 days for the membranes to dry. After peeling off, membrane weight and thickness were determined. Then, all membranes were treated using 2 M KOH solution for 24 h. The prepared membranes were labeled as indicated in Table 2.

Also, the CS-KOH membrane was prepared by adding 1 mL of 1 M KOH solution to the 10 g of chitosan (4 wt.%) solution. The KOH solution was added dropwise to the CS solution while stirring and stirred for 1 day before casting. The ZnO and POP nanoparticles were dispersed in a 2 mL aqueous mixture for 2 h before being added to the polymer blend solution. The filler loadings were in the range of 5 to 15 wt.% according to previous experience. At higher filler loadings the membrane becomes fragile, and lower loadings are too low to clearly discern any influence of the membrane composition on the monitored properties.

### 2.2. Membrane Characterization

#### 2.2.1. Membrane Thickness and Morphology

The thickness of the membranes was measured using a Mitotuyo IP65 micrometer (Mitutoyo America Corporation, Aurora, IL, USA). The average thickness in ten different regions of the membranes was taken to calculate the average value and standard deviation, for reproducibility issues. Membrane cross-section and surface properties were examined by Apreo 2C LoVac SEM (Thermo Fisher Scientific Inc., Waltham, MA, USA). Prior to analysis, membranes were coated with Au/Pd using an EMS Sputter Coater (Electron Microscopy Sciences, Hatfield, PA, USA).

#### 2.2.2. Water and KOH Uptake

Water and KOH uptake were determined using a similar method. The KOH uptake procedure was adapted from Santos et al. (2019) with a slight modification [9]. Firstly, the membranes were peeled off from the Petri dish and weighed without any further treatment ($w_{dry,KOH}$). Then, the membranes were put in 50 mL of 2 M KOH solution for 24 h. After that, membranes were taken out from the solution, washed with DI water to remove excess KOH solution, gently tapped with tissue paper to remove the water droplets on the membrane surface and weighed ($w_{wet,KOH}$).

(1) $$KOH\;Uptake\;\left(\%\right)=\frac{w_{wet,KOH}-w_{dry,KOH}}{w_{dry,KOH}}\times 100$$

In the case of water uptake, the membranes were immersed in 50 mL of DI water for 24 h and weighed ($w_{wet,i}$). A drying step was included after soaking the membrane in DI water. This weight is reported as the dry weight of the membrane ($w_{dry,i}$). The equation used to calculate water uptake of PVA-containing membranes is slightly different [18] to account for the high hydrophilic character of PVA in the final hydrophilicity of the membranes:

(2) $$Water\;Uptake\;\left(\%\right)=\frac{w_{wet,i}-w_{dry,i}}{\frac{w_{wet,i}-w_{dry,i}}{1.0}+\frac{w_{dry,i}}{1.3}}\times 100$$

where the 1.0 and 1.3 factors are the correction factors for water and PVA densities, respectively.

#### 2.2.3. Ion-Exchange Capacity (IEC)

The ion exchange capacity was determined by back titration. Briefly, the membrane was soaked in 1 M NaOH for 24 h to exchange all sites with the OH^−^ ion. The membrane was rinsed and immersed in ultrapure water (18.2 MΩ; Millipore) for 24 h to ensure the removal of residual NaOH on the membrane. The OH^−^-exchanged membranes were soaked in 0.1 M HCl for 24 h to exchange all sites with chloride ion. Then, the HCl solution was titrated with 0.1 M NaOH, and phenolphthalein was used as indicator. The NaOH solution was standardized with potassium hydrogen phthalate before titration. The blank HCl solution was also titrated with NaOH, and the concentration difference between the blank and residual HCl led to the determination of the ion exchange capacity by the following equation [11]:

(3) $$IEC\;\left(mmol/g\right)=\frac{\left(V_{blank}-V_{Sample}\right)\times C_{NaOH}}{w_{dry}}$$

where $V_{blank}$ is the titrant volume for the initial HCl solution, $V_{Sample}$ is the titrant volume for the sample solution after 24 h, $C_{NaOH}$ is the molarity of the NaOH solution and $w_{dry}$ is the weight of the dry membrane.

#### 2.2.4. Streaming Potential

The membrane surface charge was analyzed with the streaming potential via AntonPaar Surpass 3 (Anton Paar GmbH, Graz, Austria). A 0.01 M KCl solution was used to determine the surface charge, and the pH was adjusted by using 0.05 M NaOH and 0.05 M HCl solutions. All membranes were soaked in 2 M KOH solution for 24 h before analysis. The zeta potential measurement started with pH 11 by pH scan mode and decreased to pH 2 with a 0.5 pH step size.

#### 2.2.5. NaCl and KOH Permeability

**Figure 1:**
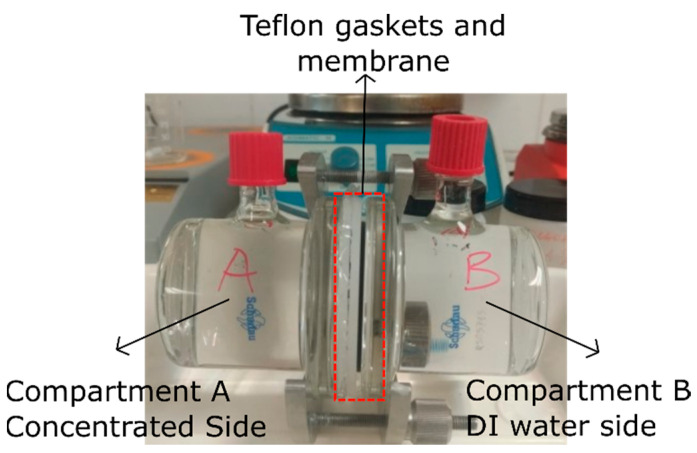
Diffusion cell.

A diffusion cell with an active area of 13.85 cm^2^ of the membrane and 120 mL of the volume of each compartment were used to determine the permeability of the membrane (Figure 1). DI water was sprayed on the surface of membrane before placing it between compartments, and a solution of 1 M NaCl was used for NaCl in the concentrated side of the cell (compartment A). The other side, compartment B, was filled with ultrapure water.

The conductivity of each compartment was measured by using HACH HQ series conductivity (Hach Company, Loveland, CO, USA) every 30 min, and the concentrations of the solutions were determined according to the calibration curve of NaCl solutions. One-and-a-half-hour data were used to determine permeability as follows [11]:

(4) $$P=\frac{\left(C_{B,t}-C_{B,0}\right)V_{B}L}{(t-t_{0})C_{A,0}A_{m}}$$

where *P* is the permeability; $C_{B,t}$ and $C_{B,0}$ are the concentrations in dilute compartment *B* at time *t* and 0, respectively; $C_{A,0}$ is the initial concentration in the feed compartment; and $V_{B}$, L and $A_{m}$ are the volume of compartment *B*, the thickness of the membrane and the active area of the membrane in the cell, respectively.

KOH permeability is determined by using the same method. A 2 M KOH solution is used instead of a 1 M NaCl solution in the concentrated side of the diffusion cell.

Also, the concentrations of several samples were validated by chloride determination using Mohr’s method. Samples were titrated with standardized 0.025 M AgNO_3_, and K_2_CrO_4_ (5 wt.%) was used as an indicator. The NaCl concentration of the solution was determined using Equation (5), and the permeability was calculated as mentioned earlier.

(5) $$C_{NaCl}=\frac{V_{AgNO_{3}}\times C_{AgNO_{3}}}{V_{sample,NaCl}}$$

where $C_{NaCl}$ and $C_{AgNO_{3}}$ are the concentrations of NaCl and AgNO_3_, respectively. The volumes of the NaCl and AgNO_3_ solutions are indicated as $V_{sample,NaCl}$ and $V_{AgNO_{3}}$.

## 3. Results

The water and KOH uptake data of membranes are given in Table 3 and compared with commercial Sustainion and Celgard 3401 membranes.

Pure CS and PVA membranes show similar trends whereby KOH uptake is higher than water uptake. The water uptake values of the CS and PVA membranes are 162% and 83.3 ± 0.6%, while the KOH uptake values are 200% and 394 ± 7%, respectively (Table 3). The CS membrane has lower hydrophilicity than PVA. Hence, increasing the CS concentration decreases the water uptake capacity of the membranes slightly. Among the different PVA-CS membranes, PVA-CS-6 is chosen for the MMM solution due to its high KOH uptake (380 ± 61%), as well as water uptake (82.3 ± 3.1%). The Sustainion membrane, which is a widely used commercial AEM membrane, was also evaluated for KOH uptake and water uptake. The KOH and water uptake values of the Sustainion membrane were found to be 198% and 169%, respectively. These values are higher than those of the PVA-CS membranes, and the operation stability of the Sustainion membrane was also superior to that of the PVA-CS membranes [30].

**Figure 2:**
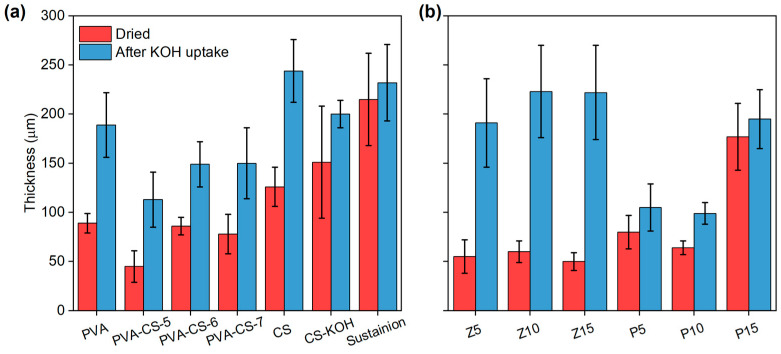
Membrane thickness before and after KOH uptake: (**a**) PVA, CS and Sustainion membranes; (**b**) ZnO- and POP added PVA-CS membranes.

**Figure 3:**
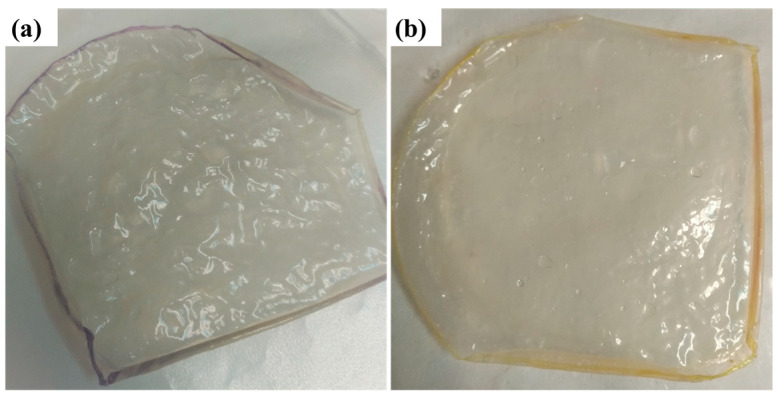
Sustainion membrane (**a**) before and (**b**) after water uptake.

To further investigate the dimensional stability of the membranes, the thickness of the membranes was measured before and after uptake. The thickness of the as-prepared membranes varies between 45 and 151 µm (Figure 2). The CS membranes result in higher thickness while the thickness of the PVA-CS membranes is below 100 µm, as expected. Although the reported thickness of the Sustainion membrane is 50 µm, its wrinkled structure results in higher thickness than that of the reported value, measured as 215 ± 47 µm (Figure 3). While the thickness of the PVA-CS membranes after KOH uptake changes between 113 and 150 µm, the addition of POP-1 and ZnO changes the thickness after KOH uptake drastically. While the thickness of the ZnO-supplemented membranes changes between 50 and 80 µm before KOH uptake, the thickness of Z10 increases from 60 µm to 223 µm after KOH uptake. On the other hand, POP-1 addition reduces the thickness change after addition. The biggest change in thickness after KOH uptake is in the P10 membrane, from 64 µm to 99 µm. Furthermore, ZnO addition lowers the membrane dimensional stability of the MMM, and POP-1 addition increases the dimensional stability after KOH uptake.

**Figure 4:**
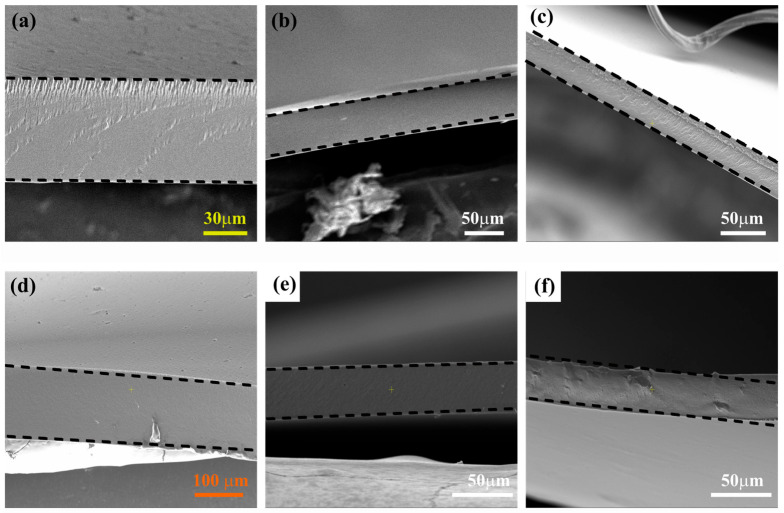
SEM images of some membranes: (**a**) PVA, (**b**) PVA-CS5 (50 wt.% CS), (**c**) PVA-CS7 (70 wt.% CS), (**d**) Z5 (5 wt.% ZnO), (**e**) Z10 (10 wt.% of ZnO) and (**f**) P5 (5 wt.% of POP-1). The black dash lines are guides indicating the thickness of the membranes.

Furthermore, the cross-section of some membranes was analyzed by SEM, and the images in Figure 4 show that all the membranes have dense structures, as expected.

**Figure 5:**
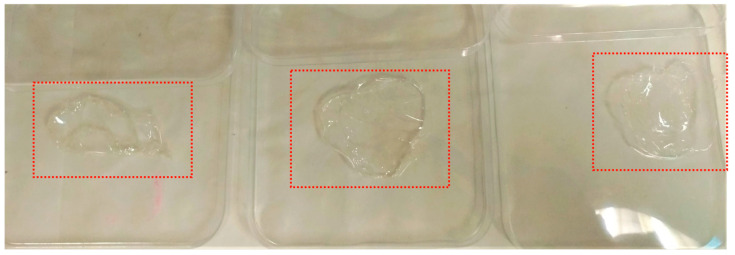
PVA-CS-6 membranes after 24 h drying. The membranes are shown in a rectangular red box to guide the eye.

The dried PVA-CS-6 membranes had wrinkles, as shown in Figure 5, making micrometer measurement for dry thickness unreliable. So, the swelling degree of these membranes could not be accurately measured, and it is not reported.

The IEC values of the CS and PVA membranes measured in this work are comparable to those reported in the literature for similar CS:PVA blend membranes. For example, Garcia-Cruz et al. reported a value of 0.27 mmol/g dry membrane [11]. The CS membranes have higher IEC values than the PVA membranes due to the larger number of OH and NH_2_ functional groups in the biopolymer backbone. Hence, the PVA-CS membrane results in high ion exchange capacity. The inclusion of ZnO and POP nanoparticles alters the properties of the PVA-CS membranes. Because of the available ion exchange sites in ZnO, the Z15 membrane has greater ion exchange capacity [31]. On the other hand, the P10 membrane has lower IEC than other membranes, except the pure-PVA membrane. The low IEC of the P10 membrane can be attributed to the bulky nature of the POPs [32], which may block available ion exchange sites.

The PVA-CS membranes have an IEC value of 0.26–0.29 mmol/g (Table 4), while the PVA membrane has a lower IEC due to fewer available sites for ion exchange than chitosan. However, increasing the CS amount from 50 wt.% to 70 wt.% does not significantly affect the IEC of the membrane. Furthermore, the IEC of the prepared membranes is consistent with the study by García-Cruz (2016) et al. [11]. The phosphonic acid group is known to increase not only the IEC performance of chitosan but also its proton conductivity [12]. PVA-based ion exchange membranes reported IEC values between 0.1 and 3.6 mmol/g depending on the procedure and crosslinking degree [33].

**Figure 6:**
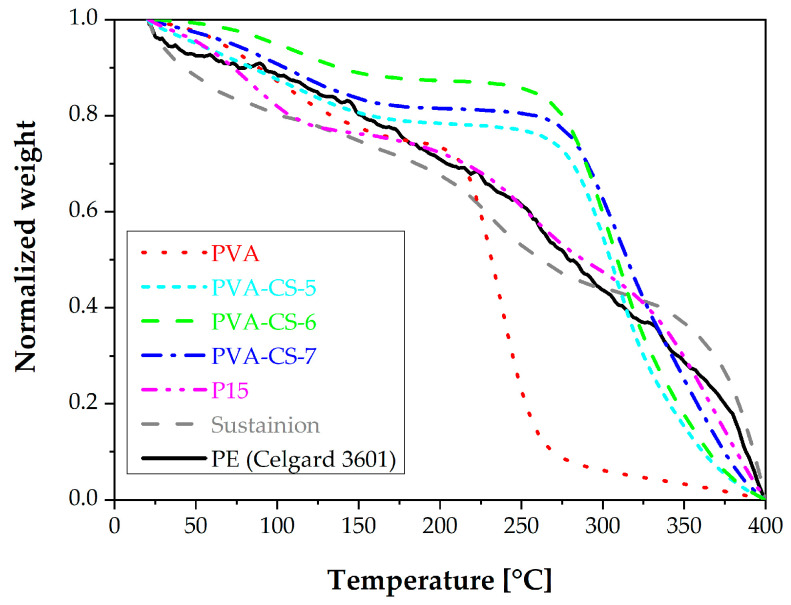
Thermogravimetric analysis of several AEMs prepared in this work (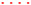 PVA, 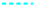 PVA-CS-5, 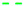 PVA-CS-6, 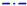 PVA-CS-7, 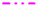 P15, 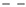 Sustainion and 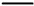 PE (Celgard 3601)).

The thermal decomposition of several of the membranes measured under a N_2_ flow of 50 mL/min from 25 to 400 °C [36] is plotted in Figure 6, with the polyethylene Celgard separator and Sustainion commercial anion-exchange membranes as references. The weight loss between 200 and 350 °C was caused by the decomposition of CS and PVA. While chitosan can degrade from 230 °C up to 400 °C, PVA degradation starts around 300 °C [37,38]. The PVA-CS membranes generally present the typical stages expected of PVA and CS blends. The CS increases the thermal stability of PVA, especially in a 60:40 wt.% ratio. That is why this blend composition was retained for the MMMs. As an example, the P15 membrane sample shows similar behavior to the unfilled PVA-CS blend membranes. This proves good compatibility between the porous filler and the polymeric matrix. The thermal decomposition increases as a function of the blend ratio and porous filler loading, as can also be observed in Table 5. This table also collects the bound water content calculated with the method reported by Franck-Lacaze et al. [36]. The values agree with the literature on AEMs and previous results obtained at the University of Cantabria for other PVA-CS membranes as alternative to Dupont and Fumatech ion exchange membranes in a polyelectrolyte membrane reactor for organic electrosynthesis at high pH [11]. The thermal decomposition of the Sustainion membrane is different than the PVA-CS membranes because of the differences in the polymer framework, made of imidazolium and polystyrene or a polyethylene substrate, respectively.

**Figure 7:**
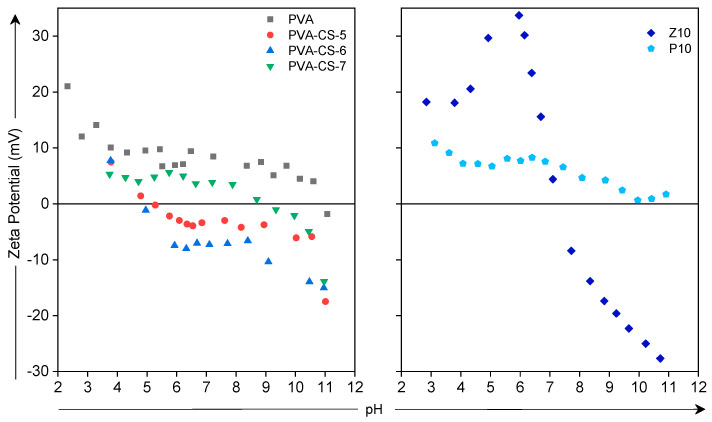
Zeta potential values of the membrane surfaces (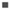 PVA, 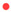 PVA-CS-5, 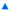 PVA-CS-6, 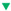 PVA-CS-7, 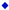 Z10 and 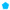 P10).

In addition to the uptake and ion exchange properties of the membranes, we determined the zeta potential of membrane surfaces in various pH values (Figure 7). While the PVA membrane exhibits positive zeta potential values in the whole pH range, the PVA-CS blend membranes show more negative surface charge at high pH values. The PVA-CS membranes show similar trends to those in the literature, as decreasing the pH of the solution resulted in an increased zeta potential result. Kyona et al. found out that various CS (content up to 40%) membranes have higher zeta potential values when the pH of the environment decreases [39]. The increase in zeta potential is the result of the protonation of the amino groups in the CS structure. The optimum amount of CS is 60 wt.% in the blend, which results in the lowest surface charge almost at all pH values, between −15.0 and 7.7 mV for pH 11 and pH 3.8, respectively. CS has positive charge below pH 6.3, whereas it is uncharged above this value [40]. At high pH, OH groups can readily deprotonate, generating a negative charge on the surface, while at low pH, NH_2_ groups can readily protonate, creating a positive charge on the surface. Thus, the surface charge becomes negative due to the domination of the OH group in the structure. Observing the MMMs with ZnO and POP nanoparticles in PVA-CS, the addition of ZnO results in a higher isoelectronic point compared with that of PVA membrane. This is attributed to the fact that ZnO nanoparticles have an isoelectric point above 8.7, showing a negative surface charge at this pH value [41]. On the other hand, adding POP particles results in a zeta potential close to that of pure PVA. This is expected due to the lack of charged sites in the structure.

Ion transport is monitored by measuring the permeability of NaCl and KOH, which could give insights into the applicability of the membranes in different applications. The NaCl permeability of the membranes changes in the range 3.45–6.05 × 10^−6^ cm^2^/s, and the KOH permeability values are higher than NaCl permeability. The addition of CS to the polymer blend leads to a more permeable membrane, with a value of 4.59 × 10^−6^ cm^2^/s. However, the KOH addition method does not significantly impact the KOH permeability of the CS membranes, as observed in Table 6. Although there is no obvious trend between the CS amount and the permeability values, all the membranes prepared in this study have higher permeability than the Sustainion membrane, which can be associated here with the high KOH and water uptake values of the PVA-CS blended membranes in Table 3. Zhong et al. demonstrated the interaction occurring between the CS functional moieties and KOH by XRD and WAXD analyses. This interaction is expected to cause CS–solvent intermediate complexes while retaining pyranose ring stacking and thus enhance swelling and uptake capacity [42].

During permeability measurements, the NaCl concentration was determined by two different methods for improved accuracy: conductivity measurement and titration. The permeability of three membranes, PVA, PVA-CS-5 and PVA-CS-6, was calculated based on the titration results and is presented in Table 7. The permeability values for PVA, PVA-CS-5 and PVA-CS-6 are 1.48 × 10^−6^, 4.98 × 10^−6^ and 3.54 × 10^−6^ cm^2^/s, respectively. When compared with the permeability values obtained via conductivity measurements (Table 6), only a slight difference observed, and the results are in good agreement. Among the three membranes, PVA-CS-5, which contains 50 wt.% of CS in the polymer solution, exhibits the highest NaCl permeability.

**Figure 8:**
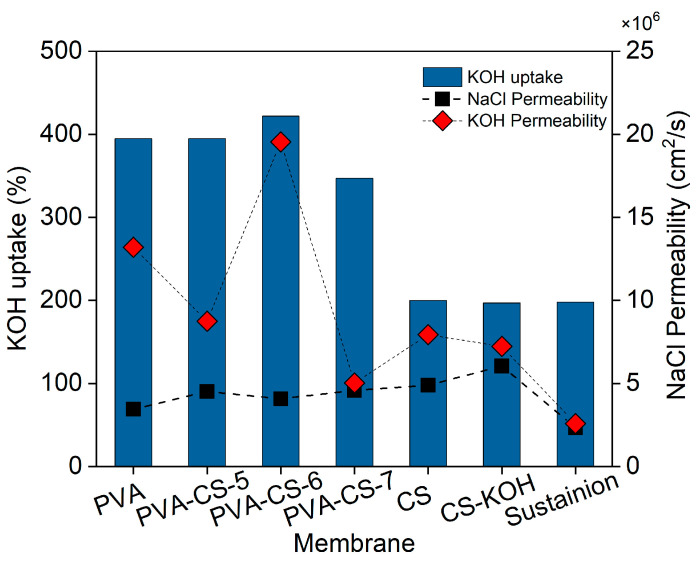
KOH uptake and permeability of the PVA-CS membranes. The commercial Sustainion membrane measured in this work as reference is also included for comparison.

The relationship between KOH uptake and permeability values is shown in Figure 8. While NaCl permeability has similar values due to the similarities in IEC of the PVA, CS and PVA-CS membranes, an optimum amount of CS results in high KOH uptake (422 wt.%) with high KOH permeability, 19.6 × 10^−6^ cm^2^/s, which is four times higher than that of the other membranes.

While the PVA-CS-6 membrane stands out among all polymer membranes, the addition of nanoparticles enhances the membrane performance. While all membranes reveal similar permeability regardless of types of nanocomposites, the Z10 membrane exhibits higher KOH uptake, 542 wt.%, and KOH permeability, 19.40 × 10^−6^ cm^2^/s, without sacrificing the permeability of the optimum PVA-CS-6 membrane. Although POP-1 addition affects both NaCl and KOH permeability in P15 significantly, KOH permeability remains below that of the Z10 membrane. Summing up, we observe that the Z10 membrane has the highest permeability and IEC, and the P10, the lowest, because of the different interaction of non-porous fillers and porous fillers within the polymer matrix, where the former disrupt the polymer chains, increasing the voids and spaces for transport, and the porosity of POP-1 allows the polymer chains to enter and reduce permeability up to a certain filler loading, where the synergy is reversed [43,44]. The high permeability values for NaCl and KOH may enable high OH^−^ ion transport and increase the efficiency of CO_2_RR electrolyzers when they are used as separators in membrane electrode assemblies [18].

## 4. Discussion

Due to their unique properties, CS and PVA membranes have gained significant attention for applications in batteries and water treatment. The hydrophilicity of PVA and the cross-linking ability of CS allow for the formation of membranes with high mechanical stability, good ion exchange properties, and selectivity for the transport of specific ions, which are crucial to rechargeable batteries such as zinc–air batteries or ion exchange membranes for water purification processes. In addition, these membranes can be modified with inorganic fillers or polymeric dopants to improve their chemical and thermal resistance, which broadens their range of applications. More generally, biopolymers such as chitosan have enormous potential in a variety of sustainable technologies, from batteries and supercapacitors to electrodialysis and ion separation processes in water desalination. Their biodegradability and low toxicity position them as environmentally friendly alternatives to conventional synthetic materials, promoting the development of more sustainable technologies in energy storage and wastewater treatment.

PVA and CS are charged polymers with different ionized sites; these functional groups behave differently according to the pH of the environment. In this study, we characterize the membranes by conducting ion diffusion experiments with salt and base solutions without adjusting the pH of solutions. The medium is always at pH 7 or higher. Under these conditions, the hydroxyl functional groups dissociate, while amino functional groups are unprotonated [45]. Hence, the hydrophilicity of PVA membranes is higher than that of CS-PVA blend membranes. Due to this higher hydrophilicity, blending CS with PVA does not affect the water uptake of the membrane significantly. Moreover, with the streaming potential, it is clearly seen that PVA membranes always have a positive surface charge below pH 11 and the surface charge slightly changes with the increase in pH because it is an uncharged polymer [45]. On the other hand, PVA-CS membranes have a lower surface charge than PVA membranes, which is due to the contribution of CS having amine functional groups. These amine groups can be protonated in acidic pH. Therefore, under acidic conditions, PVA-CS blend membranes have positive surface charge values. With the effect of functional groups in the blend, the water uptake and surface charge of PVA-CS membranes result in different NaCl and KOH permeability. The optimum PVA-CS blend has 60 wt.% of chitosan with the highest KOH permeability. PVA-CS-6 also has moderate KOH uptake, among others. Using optimum blend amounts, various ratios of ZnO and POP are embedded into the membranes. ZnO-containing membranes have high water and KOH uptake due to its hydrophilic nature.

The membranes developed in this work reach IEC values larger than the IEC reported for PVA. For instance, the PVA-CS-5 and PVA-CS-6 membranes exhibit values of 0.29 and 0.26 mmol/g, respectively, indicating an improvement in ion exchange capacity compared with pure PVA (0.13 mmol/g). On one hand, the addition of 15 wt.% ZnO further increases the IEC to 0.69 mmol/g. On the other hand, the optimum porous POP filler loading regarding IEC and ion transport is 10 wt.%. However, commercial membranes such as Sustainion (2.52 mmol/g) exhibit significantly higher IEC values, suggesting the need for further modifications to improve the performance of the developed membranes. In addition, the pure-PVA membrane shows a lower IEC value, highlighting the importance of its combination with CS to improve its properties. Here, we prove an enhancement in KOH permeability in PVA-CS blend membranes. Furthermore, we observe a trade-off relationship between water uptake and KOH permeability in PVA-CS membranes, which is consistent with literature reports on the trade-off between OH^−^ transport and ion exchange capacity (IEC) or between water and OH^−^ uptake. This behavior facilitates OH^−^ transport via the Grotthuss mechanism; thus, the result indicates the expected increase in OH^−^ conductivity. Although there is still a need to improve and investigate the applicability of PVA-CS membranes in different applications, Santos et al. (2019) showed that PVA-KOH gel membranes could perform in cyclic voltammetry and be used as OH^−^ transport membranes in a battery application [9]. The incorporation of porous and metal-containing fillers in PVA-CS membranes can open up applications such as membranes inwater treatment [46]. This work incorporates knowledge on the physicochemical and transport properties influencing membrane performance in existing or future applications.

## 5. Conclusions

Chitosan (CS) is a sustainable alternative to petroleum-based polymers investigated for different membrane applications from water treatment to fuel cells. We investigate different PVA-CS blend membranes and the influence of adding two different fillers on different physicochemical properties that are relevant in water treatment and clean energy applications. The membrane blended with 60 wt.% CS is found optimum with both the highest ion exchange capacity and permeability values of 0.26 mmol/g dry membrane and 19.54 × 10^−6^ cm^2^/s, respectively. While the addition of non-porous ZnO fillers changes the membrane behavior at all pH values, porous POP addition does not cause any change in the surface properties. Hence, ZnO addition to polymer blend results in KOH uptake of more than 500% without sacrificing the high KOH permeability of 19.40 × 10^−6^ cm^2^/s. Overall, ZnO addition to PVA-CS blend membranes has potential for OH transport membrane applications. In this light, the combination of CS and PVA seems to balance stability and ion-exchange capacity, opening up the possibility of using sustainable materials in membrane fabrication, where additional strategies are required to reach levels comparable to fossil-based commercial materials.

## Figures and Tables

**Table 2:** Polymer solutions and polymer content of the membranes.

Membrane Code	PVA Solution Concentration	CS Solution Concentration	CS	Nanomaterial	
	(wt.%)	(wt.%)	(wt.%)	Type	(wt.%)
PVA	4	-	0	-	-
CS	-	4	100	-	-
CS-KOH	-	4	100	-	-
PVA-CS-5	4	2	50	-	-
PVA-CS-6	4	2	60	-	-
PVA-CS-7	4	2	70	-	-
Z5	4	2	60	ZnO	5
Z10	4	2	60	ZnO	10
Z15	4	2	60	ZnO	15
P5	4	2	60	POP-1	5
P10	4	2	60	POP-1	10
P15	4	2	60	POP-1	15

**Table 3:** Membrane uptake properties.

Membrane	KOH Uptake (%)	Water Uptake (%)
CS	200	162
CS-KOH	197	221
PVA	394 ± 7	83.3 ± 0.6
PVA-CS-5	405 ± 10	82.3 ± 1.5
PVA-CS-6	380 ± 61	82.3 ± 3.1
PVA-CS-7	325 ± 22	81.0 ± 1.0
Z5	491	90
Z10	542	91
Z15	458 (M20)	70 (M27)
P5	298	88
P10	261	81
P15	288	85
Sustainion	198	169 80 ^1^
Celgard	85	140

^1^ Product property given by the manufacturer.

**Table 4:** Ion exchange capacity of the PVA-CS membranes prepared and measured in this study and other IEC values of PVA-CS membranes reported in the literature.

Membrane	IEC (mmol/g Dry Membrane)	Reference
CS-KOH	0.25 ^1^	This work
PVA	0.13	This work
PVA-CS-5	0.29	This work
PVA-CS-6	0.26	This work
Z15	0.69	This work
P10	0.16	This work
Sustainion	2.52 0.95	[1] [34]
Celgard	0.05	This work
CS-PVA	0.27	[11]
PVAPVA/CS	0.03 ^2^ 0.04 ^2^	[14]
PVA-CS-HDT	0.28	[35]
PCS-PVA-H 5050 Fe_2_O_3_	1.36	[10]
PCS-PVA-H 6040 Fe_2_O_3_	1.145	[25]
PVA-PGG-GP	1.52	[29]

^1^ The IEC is measured even if the membrane is totally dissolved in HCl solution. ^2^ Proton-conducting membranes.

**Table 5:** Bond water content and thermal decomposition of the membranes prepared in this study, with some commercial membranes as references.

Membrane	Water Content (%)	T_d_ (°C)	Weight Loss (%)
Celgard 3601	10.7	193	9.84
Sustainion	7.45	211	12.2
PVA	24.2	190	14.0
PVA-CS-6	14.8	222	10.0
PVA-CS-5	25.8	252	12.5
PVA-CS-7	27.5	252	13.9
P15	9.62	220	15.2

**Table 6:** Transport properties of the membranes.

Membrane	P_NaCl_ (cm^2^/s) · 10^6^	P_KOH_ (cm^2^/s) · 10^6^
CS	4.89	7.94
CS-KOH	6.05	7.23
PVA	3.45	13.20
PVA-CS-5	4.52	8.75
PVA-CS-6	4.08	19.54
PVA-CS-7	4.59	5.03
Z5	7.61	8.29
Z10	8.88	19.40
Z15	1.06	NA
P5	5.20	9.01
P10	4.94	8.38
P15	11.60	16.40
Sustainion	2.33	2.59
Celgard	1.23	1.39

**Table 7:** Permeability calculated from chloride determination.

Membrane	P_NaCl_ (cm^2^/s) · 10^6^
PVA	1.48
PVA-CS-5	4.98
PVA-CS-6	3.54

## Data Availability

The raw data supporting the conclusions of this article will be made available by the authors upon request.

## Abbreviations

The following abbreviations are used in this manuscript:

PVA	Polyvinyl alcohol
CS	Chitosan
ZO	Zinc oxide
POP	Porous organic polymer
MMM	Mixed-matrix membrane
AEM	Anion exchange membrane
PEG	Polyethylene glycol
PEO	Polyethylene oxide
CMC	Carboxymethyl cellulose
PVP	Polyvinylpyrrolidone
LSV	Linear Sweep Voltammetry
ATR-FTIR	Attenuated Total Reflectance–Fourier Transform Infrared
FESEM	Field Emission Scanning Electron Microscopy
EA	Elemental analysis
XRD	X-ray Diffraction
EIS	Electrochemical Impedance Spectroscopy
SEM	Scanning Electron Microscopy
TNM	Transference number analysis
OCV	Open Circuit Voltage
WU	Water uptake
XPS	X-ray Photoelectron Spectroscopy
IEC	Ionexchange capacity
DMFC	Direct methanol fuel cell
KHP	Potassium hydrogen phatalate
MA	Maleic anhydride
